# Size- and Voltage-Dependent Electron Transport of C_2_N-Rings-Based Molecular Chains

**DOI:** 10.3390/molecules28247994

**Published:** 2023-12-07

**Authors:** Dian Song, Jie Li, Kun Liu, Junnan Guo, Hui Li, Artem Okulov

**Affiliations:** 1 School of Materials Science and Engineering, Jiangsu University of Science and Technology, Zhenjiang 212100, China; sssdian2001@163.com (D.S.); liu_kun@163.com (K.L.); 2 Key Laboratory for Liquid-Solid Structural Evolution and Processing of Materials, Ministry of Education, Shandong University, Jinan 250061, China; guojunnan113005@hotmail.com; 3 M.N. Mikheev Institute of Metal Physics, Ural Branch of Russian Academy of Sciences, Ekaterinburg 620077, Russia; okulovartem@imp.uran.ru

**Keywords:** new-type molecular chain, electron transport, size dependence, applying the bias, first principles

## Abstract

C_2_N-ring-based molecular chains were designed at the molecular level and theoretically demonstrated to show distinctive and valuable electron transport properties that were superior to the parent carbonaceous system and other similar nanoribbon-based molecular chains. This new -type molecular chain presented an exponential attenuation of the conductance and electron transmission with the length. Essentially, the molecular chain retained the electron-resonant tunneling within 7 nm and the dominant transport orbital was the LUMO. Shorter molecular chains with stronger conductance anomalously possessed a larger tunnel barrier energy, attributing to the compensation of a much smaller HOMO–LUMO gap, and these two internal factors codetermined the transport capacity. Some influencing factors were also studied. In contrast to the common O impurity with a tiny effect on electron transmission of the C_2_N rings chain, the common H impurity clearly improved it. When the temperature was less than 400 K, the electron transmission varied with temperature within a narrow range, and the structural disorder deriving from proper heating did not greatly modify the transmission possibility and the exponentially decreasing tendency with the length. In a non-equilibrium condition, the current increased overall with the bias but the growth rate varied with size. A valuable negative differential resistance (NDR) effect appeared in longer molecular chains with an even number of big carbon–nitrogen rings and strengthened with size. The emergence of such an effect originated from the reduction in transmission peaks. The conductance of longer molecular chains was enhanced with the voltage but the two shortest ones presented completely different trends. Applying the bias was demonstrated to be an effective way for C_2_N-ring-based molecular chains to slow down the conductance decay constant and affect the transport regime. C_2_N-ring-based molecular chains show a perfect application in tunneling diodes and controllable molecular devices.

## 1. Introduction

Mostly, the electron transport of molecular wires decays exponentially with length but the structural stability also decreases rapidly with the chain elongation, limiting their applications [1,2,3]. Therefore, exploring new-type molecular wires with controllable conductance decay and good structural stability to understand electron transport [4,5] and establish well-defined structure–property relationships [6,7] has widely attracted the academic and industrial communities in fields such as field-effect transistors, logic circuits, memory and storage devices [8,9,10].

**Figure 1:**
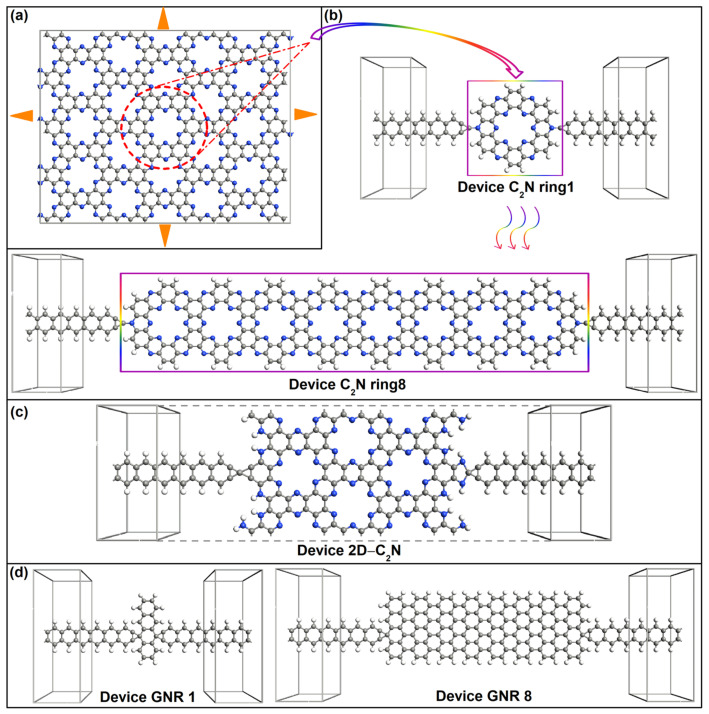
(**a**) Geometry of C_2_N monolayer.The unit cell is indicated by the red dashed circle and the corresponding. Molecular electrostatic potential is shown in Figure S1 of Supporting Information (SI) [17]. (**b**–**d**) Structure schematic of C_2_N-ring-chain-based devices, 2D-C_2_N-based device and GNR-based device, respectively.

Recently, C_2_N, which is a new two-dimensional (2D) material with a periodic porous structure, was successfully synthesized [11]. In its structure, sp^2^-hybridized carbon (C) rings are connected through two face-to-face nitrogen (N) bridges that are distributed in a honeycomb lattice, and six electronegative N atoms constitute one periodic hole (see Figure 1a) [12]. Such a unique structure exhibits extremely high thermal stability due to the strong covalent bonding framework formed by the C-C and C-N atom pairs [13]. C_2_N displays tunable semiconductor properties and presents a high on/off ratio of ∼107 as a field effect transistor [14]. Thus, extensive research about properties such as thermal conductance [15], photoelectronic properties [16], mechanical properties [17], adsorption of molecules [18,19], electrochemical properties [20,21], and gas and impurity separation [22] has been conducted, showing promising applications in the semiconductor [23], nano/optoelectronics [24], sensing technology [25], photovoltaics [26], energy [27], biological [28] and medical industries [29]. However, few studies have been focused on the electrical performance of C_2_N at the molecular scale.

Based on the excellent structural stability and electrical properties of 2D C_2_N, we theoretically designed a new-type molecular wire, which was one side-by-side single-ring molecular chain from the C_2_N monolayer, and studied the equilibrium and non-equilibrium electron transport properties, including the conductance decay and current–voltage characteristics. The corresponding structure–property relationship and inner electron transmission mechanism are well explained.

## 2. Results and Discussion

The new-style molecular wire was composed of parallel-connected single hexagonal C_2_N rings taken from a C_2_N monolayer (see Figure 1a). The length of one single C-N garland (denoted as a C_2_N ring here) was defined as one unit length. Eight lengths (i.e., 1–8 C_2_N rings) of molecular wires were studied. Figure 1b exhibits the schematic view of C_2_N-ring-chain-based devices, where the molecular wire was sandwiched between four-atom-wide graphene nanoribbon (GNR) electrodes with a tip C atom as the linked atom. On each side, the tip electrode atom was symmetrically bonded to one N atom on the outer edge of the C_2_N ring. Molecular devices were denoted as device C_2_N ring i (i = 1–8) depending on the number of C_2_N rings.

In addition, we also provide the parent material 2D-C_2_N and graphene nanoribbon (GNR)-based chains with eight lengths as a comparison. Corresponding device structures are shown in Figure 1c,d, which all have the same connection style and electrodes to C_2_N-ring-molecular-chain-based devices. One column of C rings is defined as one unit length for GNR. Correspondingly, the device with the parent material is written as device 2D C_2_N and GNR-based devices are written as device GNR i (i = 1–8) depending on the number of the unit length.

### 2.1. Equilibrium Conductance Decay and Electronic States

**Figure 2:**
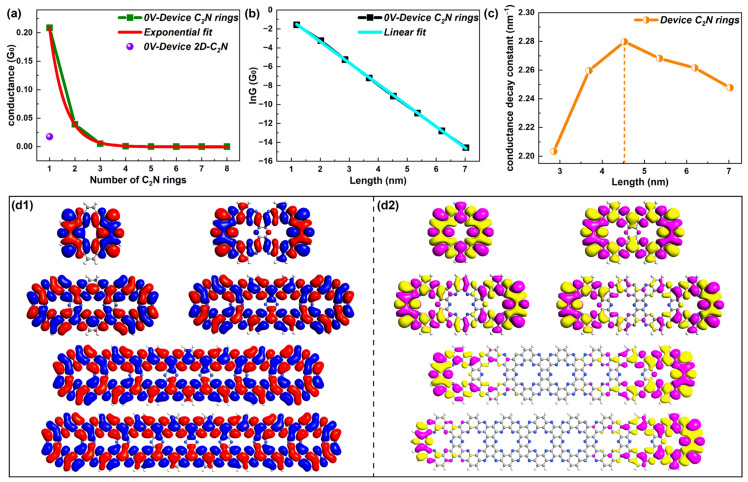
(**a**) Equilibrium quantum conductance—number of C_2_N rings curve with an exponential fit and conductance of 2D-C_2_N-based device. (**b**) lnG as a function of length with a linear fit. (**c**) Changes in the conductance decay constant within the length range of [2.85, 7] nm. (**d1**,**d2**) LUMOs and HOMOs for representative device C_2_N rings 1, 2, 3, 4, 7 and 8, respectively.

To explore the electron transport of new-type molecular wires, first, Figure 2a presents the equilibrium quantum conductance of the device 2D C_2_N and the conductance of the device C_2_N rings changing with the number of C_2_N rings with an exponential fit. Obviously, the conductance of the shorter C_2_N ring chains was stronger than the original material 2D C_2_N. The conductance attenuation with the length of a C_2_N ring chain (L) satisfies the typical equation [30]:

(1) G=Aexp(−βL)

where *β* is the decay constant, which is calculated from the slope of the plot of logarithmic conductance (ln*G*) vs *L*. More clearly, the ln*G*–length curve with a linear fit is also given in Figure 2b. *A* is a constant related to the interaction between the molecular wire and electrodes, reflecting the contact resistance.

Figure 2c gives the *β* values for different molecular lengths. Noticeably, *β* varied with length and started to drop when the length was over 4.5 nm (five rings). However, *β* changed little (from 2.20 to 2.24 nm^−1^) in the length range [3 nm, 7 nm] and the C_2_N ring chain always maintained an exponentially attenuated conductance within 7 nm, indicating that the C_2_N ring chain possessed resonant tunneling as the dominant transport mechanism within 7 nm. This was quite different from traditional molecular junctions, which took the direct tunneling regime as the dominant transport mechanism with lengths less than 4 nm and presented an obvious decreased *β* with lengths over 4 nm, evidencing a transition between the tunneling and hopping mechanism [31,32].

Further, the focus of the following research was the electronic structures of molecular rings, mainly highlighting the electronic states in the transport process, which is closely linked to transport decay [33,34]. The eigenstates of the molecular projected self-consistent Hamiltonian (MPSH) can be considered as molecular orbitals renormalized by the molecule–electrode interaction [35], giving a visual description of the molecular electronic structure [36]. Thus, frontier molecular orbits (LUMOs and HOMOs) are given. Representative states of LUMOs and HOMOs are presented in Figure 2d1,d2, respectively. Others can be found in Figure S2 of the SI. The electronic states of LUMOs were delocalized for C_2_N ring chains for all lengths, while the wavefunctions started to be localized and were mainly distributed in the rings on both sides from device C_2_N ring 3. This indicates stronger electronic states of the LUMO, in stark contrast to the HOMO. Thus, the dominant transport orbital was the LUMO for C_2_N-ring-chain-based molecular devices in the tunneling mechanism.

### 2.2. Mechanisms: Electron Transmission, HOMO–LUMO Gap and Tunnel Barrier Energy

**Figure 3:**
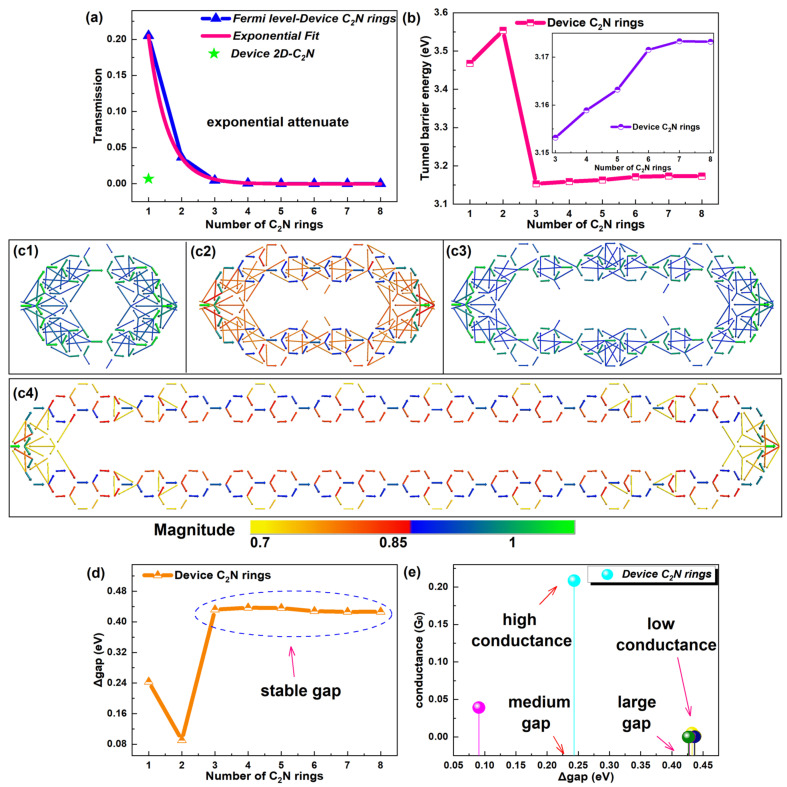
(**a**) Equilibrium electron transmission for E_F_ number of C_2_N rings curve with an exponential fit and the transmission of a 2D-C_2_N-based device. (**b**) Tunnel barrier energy as a function of number of C_2_N rings in [1, 8], and the inset shows the corresponding changes in a narrower range of [3, 8]. (**c1**–**c4**) Transmission pathways of representative device C_2_N rings 1, 2, 3 and 8, respectively. (**d**) HOMO–LUMO gap as a function of number of C_2_N rings. (**e**) Conductance as a function of HOMO–LUMO gap of C_2_N rings.

The transmission at the Fermi level (E_F_) expresses the transport capacity of molecular devices [37]. Thus, Figure 3a shows the trend of the transmission coefficient at E_F_ changing with the number of C_2_N rings and the transmission of the device 2D-C_2_N. Similar to the conductance, the electron transmission coefficient at E_F_ was much larger for shorter device C_2_N rings in contrast to the parent-material-based device, signifying stronger electron transport abilities of shorter C_2_N-ring-based chains. The electron transmission probability took on an exponential decline, directly explaining the reduction in the conductance with length. On a deeper level, the tunnel barrier energy, i.e., the energy difference between the dominant transport orbital and the electrode Fermi level, is the internal determinant for the electron transport of molecular devices [38]. Usually, the tunnel barrier energy of the molecular wire would increase as the length increases, essentially causing the conductance decay [39]. Unexpectedly, the tunnel barrier energy of the C_2_N ring chains did not show the overall uptrend with length (see Figure 3b). Device C_2_N rings 1 and 2 with stronger transport capacity actually possessed higher tunnel barrier energy compared with the longer devices. When the number of rings grew from 1 to 2 or 3 to 8, the tunnel barrier energy followed the general rule.

Furthermore, such an abnormal phenomenon of the intrinsic factor is directly reflected in the transmission pathways, which are in essence transition paths of electrons between atoms [40]. Transmission pathways of representative devices are exhibited in Figure 3c1–c4. Others are shown in Figure S3 of the SI. Specifically, the thickness of the arrow indicates the magnitude of the local transmission between each pair of atoms, and the arrowhead and the color designate the direction of the electron flow [40]. Obviously, electrons transferred along little C/C_2_N rings up and down the outer edges, not passing the joint rings. In particular, stronger transmission appeared in little C_2_N rings, judging by the color and width of the transmission arrows. No matter whether the number increased from 1 to 2 or 3 to 8, the transferring arrows gradually reduced and became thinner, indicating that the electron transport weakened, but the transmission power of device C_2_N ring 3 was stronger than device C_2_N ring 2. All these were consistent with the tendency of the tunnel barrier energy.

To investigate the anomalous behavior, the tendency of the HOMO–LUMO gap with the number of C_2_N rings was studied (see Figure 3d). Shorter chains presented smaller gaps. Larger and stable gaps were found for longer chains. The conductance as a function of the gap is also provided in Figure 3e. For the new-style chain, a large gap did not bring about strong conductance, and high conductance occurred in the chain with a medium gap. Generally, a wider HOMO–LUMO gap produced higher conductance [1]. Clearly, a narrower gap of device C_2_N rings 1 and 2 compensated for their larger tunnel barrier energy. These two internal factors codetermined the conductance and transport capacity of the C_2_N-ring-chain-based devices.

### 2.3. Comparisons with Other Similar Systems and Influencing Factors

**Figure 4:**
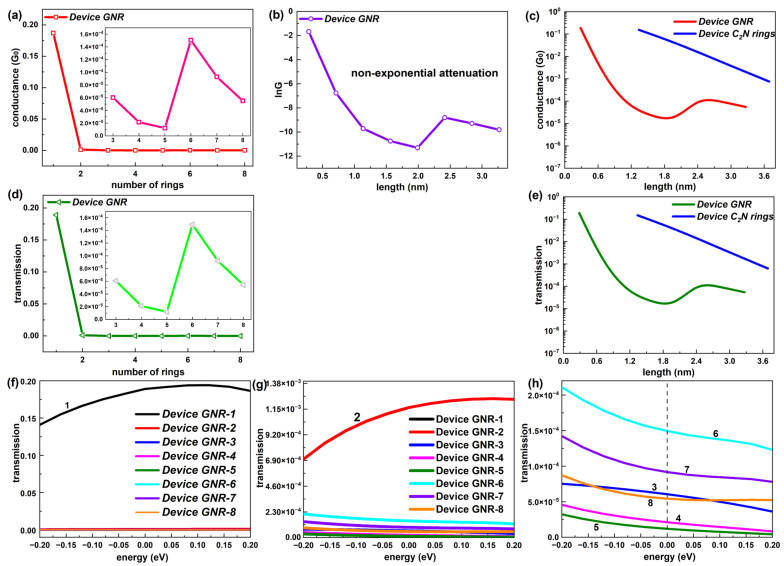
(**a**) Conductance at E_F_ as a function of number of rings (1–8) for GNR-based devices. The inset shows the number range [3, 8]. (**b**) lnG as a function of length for GNR-based devices. (**c**) Comparison of conductance between C_2_N-ring-chain-based devices and GNR-based devices changing with the length. (**d**) Electron transmission at E_F_ as a function of number of rings (1–8) for GNR-based devices. The inset shows the number range [3, 8]. (**e**) Comparison of electron transmission at E_F_ between C_2_N-ring-chain-based devices and GNR-based devices changing with the length. (**f**–**h**) Transmission spectrum around E_F_ of GNR-based devices in transmission ranges [0, 0.2], [0, 1.38 × 10^−3^] and [0, 2 × 10^−4^], respectively.

For comparison, Figure 4 gives the equilibrium electron transport performance of the GNR-based chains with different lengths. Unlike the device C_2_N rings, the device GNR presented a fluctuating tendency of conductance with the number of columns of C rings (called the number of rings), as shown in Figure 4a and the corresponding inset (shown in a narrower range). For more clarity, the ln*G*–length graph provided in Figure 4b and Figure 4c exhibits the comparison of the conductance as a function of the length for C_2_N-ring- and GNR-based chains on a log scale. Obviously, the conductance of the device GNR showed non-exponential attenuation with the length of the chain. Furthermore, the device C_2_N had a distinct advantage over the device GNR on the conductance strength.

Consistent with the conductance, the electron transmission coefficient at E_F_ of the GNR-based chain took on an undulating state that varied with the number of rings, which can be found in Figure 4d. A contrast of the transmission–length relationship between the device C_2_N rings and the device GNR is also presented on a log scale in Figure 4e. It follows that such a trend was also not an exponential decline and the transmission coefficient of the device C_2_N rings was far higher than the device GNR at the same length. Thus, the new-type C_2_N-ring-based chains possessed better electronic transport capacity compared with the GNR-based chains.

Further, Figure 4f,g exhibit the comparison of the transmission spectrum within the energy interval [−0.2 eV, 0.2 eV], i.e., near E_F_ among the GNR-based chains with different lengths. From Figure 4f, the electron transmission of the shortest GNR-based chain was significantly stronger than other lengths of GNR chains in the whole energy range. Device GNR-2 also displayed a much larger transmission coefficient than longer GNR chains near E_F_, according to the inset of Figure 4f. For these two shortest GNR-based chains, the transmission probability increased gradually from −0.2 eV to 0.2 eV. The other six lengths of GNR-based chains all presented quite low electron transmission probabilities, which were distributed in the same order of magnitude (10^−5^). Unlike C_2_N-ring-based chains with the law of decline, there were no specific rules in the relative strength of the electron transmission among these six GNR-based chains (GNR-3 to GNR-8). Different from the two shortest GNR-based chains, these six longer chains all showed slowly decreasing transmission values from −0.2 eV to 0.2 eV. Overall, the C_2_N-ring-based chains demonstrated a more regular transmission spectrum with the length.

**Figure 5:**
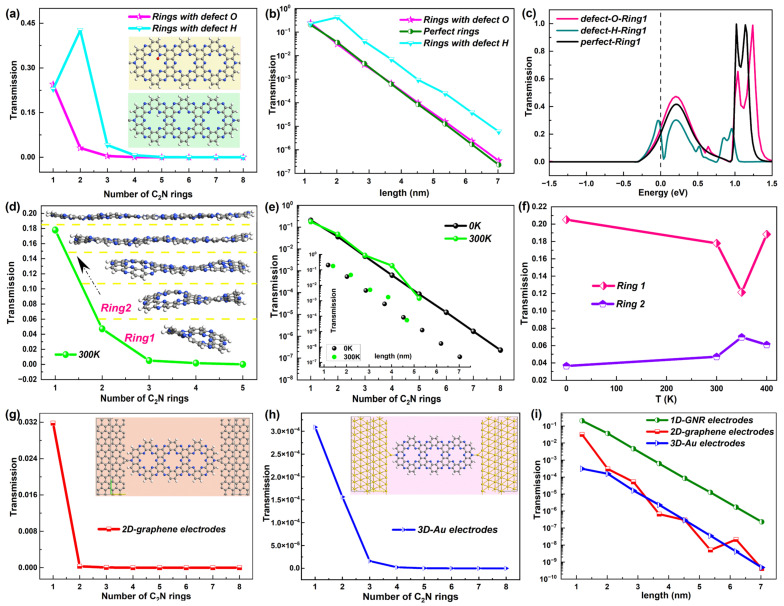
(**a**) Electron transmission at E_F_ of the C_2_N ring chains with O and H impurities, changing with the number of C_2_N rings. The insets show the positions of two such impurities. (**b**) Comparison of electron transmission at E_F_ of the C_2_N ring chains with no defect and O and H impurities, changing with the length of the chains. (**c**) Electron transmission spectrum around E_F_ of device C_2_N ring 1 with no defect and O and H impurities. (**d**) Electron transmission at E_F_ as a function of the number of C_2_N rings at 300 K. The insets show the corresponding 5 structures of the C_2_N ring chains at 300 K. (**e**) Comparison of electron transmission at E_F_ of C_2_N rings at 0 K and 300 K changing with the number of C_2_N rings and the length (shown in the inset), respectively. (**f**) Electron transmission at E_F_ of device C_2_N rings 1 and 2 as a function of the temperature T. (**g**,**h**) Electron transmission at E_F_ as a function of the number of C_2_N rings for the C_2_N-ring-chain-based devices with 2D graphene electrodes and 3D Au electrodes, respectively. The insets show the device structures. (**i**) Comparison of electron transmission at E_F_ of C_2_N ring chains based on 3 different electrodes changing with the length of the chains. For clarity, all comparisons are shown on a logarithmic scale.

In addition, some factors were also considered to investigate the corresponding influence on the electron transport of the new-type molecular chain, including the defects, disorder, temperature and electrode materials. The results of the synthesis and characterization show that there were varieties of point defects in the C_2_N-based materials, mainly including the C and N vacancies, as well as the substitutional and interstitial impurities of C, N, O and H atoms [11]. Here, we considered two common defects, that is, H and O impurities in the big ring, which were shown to possess relatively lower formation energies in defective C_2_N-based materials [16]. For each length of the C_2_N ring chain, one defect atom (H or O) was considered here. The insets of Figure 5a exhibit the positions of H or O impurities in the C_2_N ring chain.

To study the influence of the defects on electron transport, first, Figure 5a presents the electron transmission at E_F_ changing with the number of C_2_N rings for these two kinds of defective C_2_N ring chains. One obvious and unexpected increase could be found in device C_2_N ring 2 with a defect H atom, which broke the decreasing trend of electron transmission with the length of the perfect C_2_N ring chain. However, as the length continued to increase, the electron transmission still went down with the length for the C_2_N ring chain with an H impurity. As a result, the value of each C_2_N ring was higher than the perfect C_2_N rings but still showed an exponential decline with the length, which could be seen in the comparison of electron transmission at E_F_ changing with the length among the devices with no defects and the two defects (Figure 5b). The C_2_N-ring-based chain with an O impurity presented a similar electron transmission value and exponential downward trend to the perfect C_2_N-ring-based chain, indicating the slight influence of the O impurity on the electron transmission at E_F_ of the C_2_N-ring-based chain. Further, the electron transmission spectrum around E_F_ is given. Figure 5c shows the comparison of the spectrum for device C_2_N ring 1 with no defect and H and O impurities, while others are exhibited in Figure S4 of the SI. The O impurity also slightly affected the distribution and value of electron transmission near E_F_ but changed the height and position of the transmission peaks for larger energies. In contrast, the H impurity greatly modified the transmission curve around E_F_.

When the temperature rises, structural distortions, deformation and disorder occur in C_2_N rings, especially shorter chains, and may affect the electron transport properties. When the temperature rose to 300 K, significant structural distortions could be observed in rings 1–3 with ups and downs, which can be seen in the insets of Figure 5d. As the length increased, the C_2_N ring chain showed less structural fluctuation, which is also consistent with the stable structure of 2D-C_2_N [25,41], but possessed a little higher electron transmission coefficient at E_F_ than the rings at 0 K, and one prominent enhancement in the transmission value appeared in ring 4, which is reflected in Figure 5e. Furthermore, when the C_2_N ring chain was at 300 K, the length increased slightly compared with the chain at 0 K. However, such structural disorder originating from a higher temperature did not change the exponentially decreasing tendency of electron transmission at E_F_ with the length.

To further investigate the effect of the temperature on the electron transport, Figure 5f displays the electron transmission at E_F_ as a function of the temperature T for C_2_N ring 1 and C_2_N ring 2 as representatives based on the larger structural changes of shorter chains. They do not show the monotonical changing trend of the electron transmission coefficient at E_F_ with T. For the shortest C_2_N ring 1, the electron transmission coefficient at E_F_ decreased with T until 350 K and then increased again. Differently, C_2_N ring 2 showed enhanced electron transmission at E_F_ until 350 K and then decreased again, but the changing range of electron transmission was not significant and was smaller than that of C_2_N ring 1. No matter how the temperature changed, the electron transmission possibility at E_F_ of ring 1 was bigger than that of C_2_N ring 2 and the value did not vary greatly with T for the two rings.

Furthermore, as an important part of the molecular device, the electrodes may greatly affect the electron transport properties. Therefore, we chose different types of materials as the electrodes to study such an influence, namely, 2D graphene and 3D Au electrodes in contrast to the original 1D GNR electrodes. The corresponding structures of the two devices are presented in the insets of Figure 5g,h, which also show the electron transmission at E_F_ changing with the number of C2N rings. It is not hard to find the sharp drop in the electron transmission value with the number of C_2_N rings in these two devices. For clarity, Figure 5i presents the comparison of the electron transmission at E_F_ of the C_2_N ring chains based on these three different electrodes changing with the length of the chains on a logarithmic scale. The transmission possibility of 3D-Au-electrode-based device maintained the exponential attenuation with length with a similar speed to the 1D-GNR-based device but was much weaker than the same length of C_2_N ring chain. The outcome was different for the 2D-graphene-based device. The electron transmission still became weaker with increasing length but was no longer strictly exponential. When the length of the C_2_N ring chain was the same, the transmission coefficient was always noticeably lower than the 1D-GNR-based device, but sometimes a little higher than the 3D-Au-based device.

### 2.4. Non-Equilibrium Electron Transport Properties: IV Characteristics

**Figure 6:**
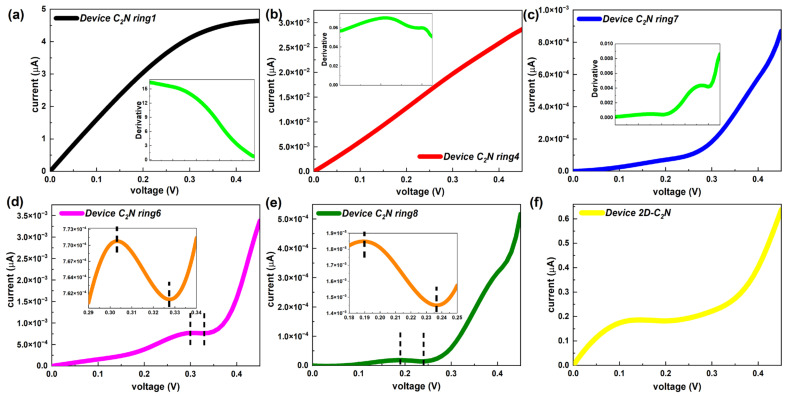
(**a**–**c**) I–V curves of representative device C_2_N rings 1, 4 and 7, respectively. Each inset shows the first derivative of corresponding I–V curve. (**d**,**e**) I–V curves of device C_2_N rings 6 and 7, respectively. I–V curves within the specific bias interval are exhibited in the insets. (**f**) I–V curve of the device 2D-C_2_N.

Non-equilibrium electron transport properties were also studied for the C_2_N ring chains. The current–voltage characteristics are related to practical and important non-equilibrium performance [42]. Figure 6 presents typical current–voltage curves with derivatives at biases, representing changing rates. Others are given in Figure S5 of the SI. Overall, the current of the C_2_N-ring-based chains increased with the voltage but showed different rates based on different lengths. When the number of big carbon–nitrogen rings did not exceed three, the growth rate of the current went down monotonically with increased voltage. The current of device C_2_N rings 4 and 5 tended to grow linearly at a rate with a gentle variation. When the number exceeded five, the speed of the current rose with voltage. Moreover, at the same bias, the growth rate of current decreased as the length increased.

Remarkably, as the length of the C_2_N ring chain increased, the I–V curve no longer strictly maintained the increasing trend and started to show a negative differential resistance (NDR) effect, which is indispensable for several electronic components, such as the Esaki and resonant tunneling diodes [4]. A slight NDR feature only appeared in the longer devices with an even number (6 and 8) of C_2_N rings. As presented in Figure 6d,e, device C_2_N ring 6 showed a peak-to-valley current ratio (PVCR) of 1.01 in the bias range [0.3 V, 0.33 V], while device C_2_N ring 8 had a PVCR of 1.27 between 0.19 V and 0.24 V. Here, the PVCR is equal to the ratio of the maxima and the minima of the current in the voltage range where the current drops [43]. It follows that NDR would enhance, that is, the voltage range where NDR occurs would enlarge and the PVCR would rise, as the number of C_2_N rings increases. In contrast, the I–V performance of the device 2D-C_2_N showed a non-linear increasing trend and had a long plateau of slow growth, which was more like the overall trends of longer C_2_N-ring-chain-based devices but did not show a valuable NDR effect.

**Figure 7:**
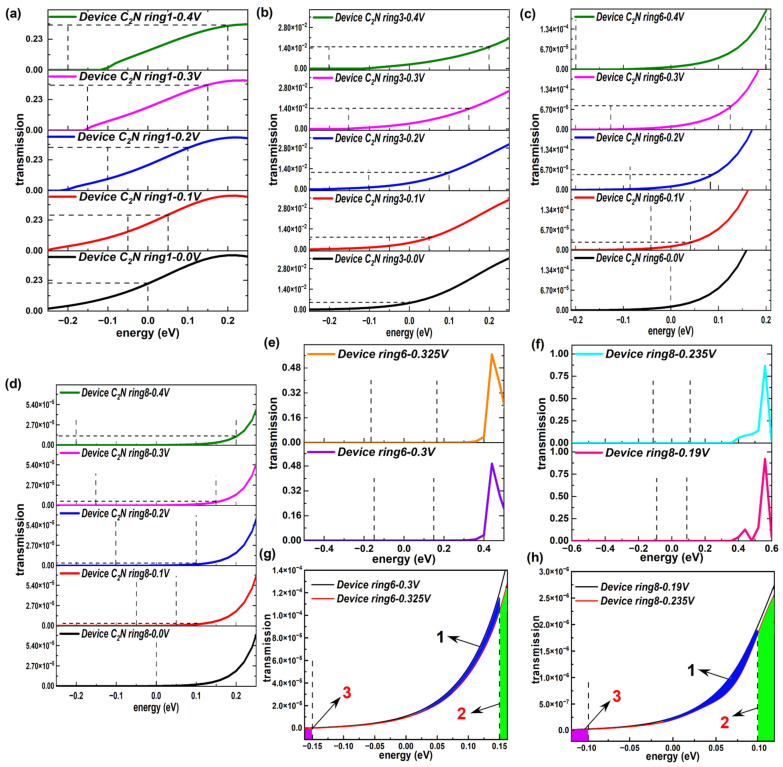
(**a**–**d**) Transmission spectrum within narrower energy ranges at 0 V, 0.1 V, 0.2 V, 0.3 V and 0.4 V of representative device C_2_N rings 1, 3, 6 and 8, respectively. (**e**) Transmission spectrum at 0.3 V and 0.325 V of device C_2_N ring 6. (**f**) Transmission spectrum at 0.19 V and 0.235 V of device C_2_N ring 8. (**g**,**h**) Transmission spectrum in (**e**,**f**) within narrower energy ranges.

Here, the current was calculated using the Landauer–Büttiker formula, indicating that the current through devices was inseparable from the transmission coefficients of the devices. To explain the current–voltage characteristics of the C_2_N-ring-based chain, Figure 7 presents the transmission functions of the device C_2_N rings under representative biases. In our calculations, the average Fermi level, which was the average chemical potential of left and right electrodes, was set as zero. The current was determined using the integral area of the transmission curve within the bias window, i.e., [−V/2, +V/2] [44], shown as dashed lines. For all C_2_N-ring-based devices, 0.1 V, 0.2 V, 0.3 V and 0.4 V were selected as representative biases. Actually, there was just the left side of the transmission peak inside each bias window for all C_2_N-ring-based devices and the summit of the transmission peak was far away from E_F_ for most C_2_N-based devices, except the shortest device C_2_N ring 1, which is demonstrated by the transmission functions under four representative biases within larger energy intervals in Figure S6 of the SI. Therefore, to display the changes of the integral area within the bias window more clearly, transmission curves under non-equilibrium conditions are presented in narrower energy ranges. Several typical devices (device C_2_N rings 1, 3, 6 and 8) are shown in Figure 7a–d and others are displayed in Figure S7 of the SI. There were differences in the growth rates of transmission curves between C_2_N-based devices from −0.2 eV to 0.2 eV. Apparent enlargement can be found in the integral area of the transmission curve inside the bias window for these devices as the bias increased based on the variations in the height and width of the specific area (marked with a dashed line in the figure), which explains the overall increasing trend of the I–V curves well.

However, there was a noted drop in the current emerged over a small voltage interval for device C_2_N ring 6 and device C_2_N ring 8. To clearly explain the origins of the NDR effect appearing in the longer devices with an even number of C_2_N ring chains, we first provide the transmission functions in larger energy ranges under the voltages at both ends of the decreasing voltage intervals (0.3 V and 0.325 V for device C_2_N ring 6, 0.19 V and 0.235 V for device C_2_N ring 8), as displayed in Figure 7e,f. Similar to the typical biases above, under the new biases, the summits of the transmission peaks still remained away from E_F_. Because the peak value was far larger than the coefficient at E_F_, the changes in the area inside the bias window could not be directly observed. Thus, each energy range on display was narrowed to a minimum (see Figure 7g,h), corresponding to the selected voltages, i.e., [−0.325/2, 0.325/2] for device C_2_N ring 6 and [−0.235/2, 0.235/2] for device C_2_N ring 8. The integral area of the transmission peak under 0.325 V was smaller than that under 0.3 V by comparing the area 1 sandwiched between curves and the sum of extra areas 2 and 3, as judged by a huge order of magnitude difference between the horizontal and vertical coordinates. Areas 1, 2 and 3 are marked with different colors in Figure 7g,h. The same was true for device C_2_N ring 8. Therefore, the NDR effect in essence emerged from the reduction in the transmission peaks.

### 2.5. Non-Equilibrium Electron Transport Properties: Conductance–Voltage Relations

**Figure 8:**
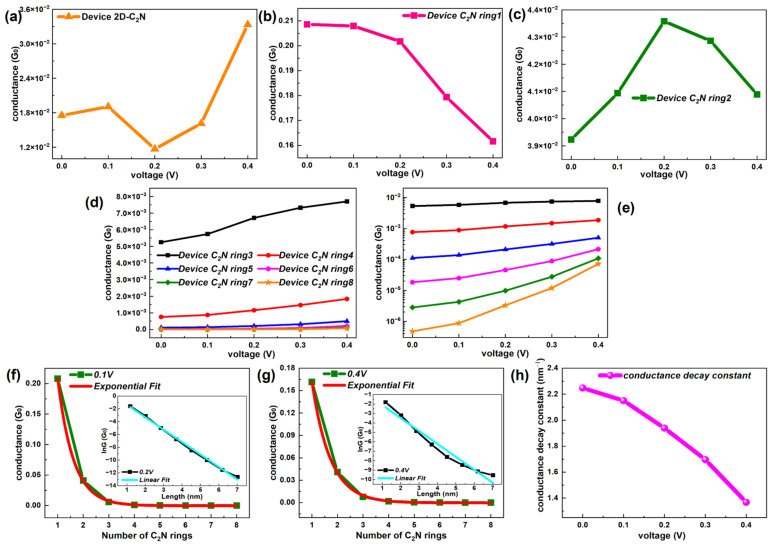
(**a**–**c**) Conductance–voltage functions of device 2D-C_2_N, device C_2_N ring 1 and device C_2_N ring 2, respectively. (**d**,**e**) Comparison of the conductance changing with bias for device C_2_N rings 3, 4, 5, 6, 7 and 8 on linear and log scales, respectively. (**f**,**g**) Conductance as a function of number of C_2_N rings with an exponential fit at 0.1 V and 0.4 V, respectively. Each inset exhibits the corresponding lnG as a function of length with a linear fit. (**h**) Changing trend of the conductance decay constant with voltage.

Non-equilibrium changes in the conductance were associated with the length and voltage. Relative relationships for the device 2D-C_2_N and device C_2_N rings are exhibited in Figure 8. Taking significant differences in conductance values into consideration, the conductance–voltage curve is given separately for the device 2D-C_2_N, device C_2_N ring 1 and device C_2_N ring 2, corresponding to Figure 8a–c, and the other six C_2_N rings-based chains are compared on a linear and log scale respectively, corresponding to Figure 8d,e. As observed, the conductance fluctuated with the bias and the values were relatively small under low biases for the parent-material-based device, which was much weaker than the two shortest C_2_N-ring-chain-based devices under any voltage.

As the bias enhances, the two shortest C_2_N-ring-based chains show peculiar conductance–voltage characteristics, which did not show any regularity in the length. The conductance of device C_2_N ring 1 dropped with the increased bias, while one single peak appeared in the conductance–voltage curve of device C_2_N ring 2. Different from the above two, the longer six C_2_N-ring-chain-based devices presented regularly enhanced conductance with voltage and the growth accelerated in larger biases, which was more prominent in longer chains. However, at each bias, the conductance still decayed with the increasing length.

To investigate the influence of the voltage on the trend of the conductance changing with the length, Figure 8f,g and Figure S8 in the SI show the conductance–length curve at several biases. When the voltage was applied, the conductance of the C_2_N-ring-chain-based devices still decayed rapidly with the length, but the trend gradually deviated from the exponential fit and the extent of this deviation deepened as the voltage increased. Further, Figure 8h gives the conductance decay constant (β) as a function of the voltage. One significant drop can be observed in the conductance decay constant with the increased voltage, suggesting a transition between resonant tunneling and the intrachain hopping mechanism under the bias. This provides an effective way for the C_2_N ring chain to control the attenuation speed of the conductance with the length or to affect the inner electron transport regime.

## 3. Computational Methods

The simulated electronic devices were constructed in Virtual NanoLab within a supercell with over 15 Å of vacuum space to allow for electrostatic interactions to decay for a system. All structures were structurally optimized before calculating the electron transport properties. All calculations used the first principles theory based on density functional theory (DFT) and non-equilibrium Green’s function (NEGF) and were performed in the Atomistix Toolkit (ATK) [45,46]. Numerical LCAO basis sets and norm-conserving pseudopotentials were adopted. For high accuracy, GGA-PBE formulation was selected and the K-point sampling was set to 1 × 1 × 100. The double-zeta plus polarization (DZP) basis for all atoms was adopted. The density mesh cut-off for the electrostatics potential was 75 Ha and the electron temperature was set as 300 K. Structural optimizations used the quasi-Newton method until all residual forces on each atom were smaller than 0.05 eV/Å. The convergence criterion for the total energy was set to 10^−5^ via the mixture of the Hamiltonian.

**Figure 9:**
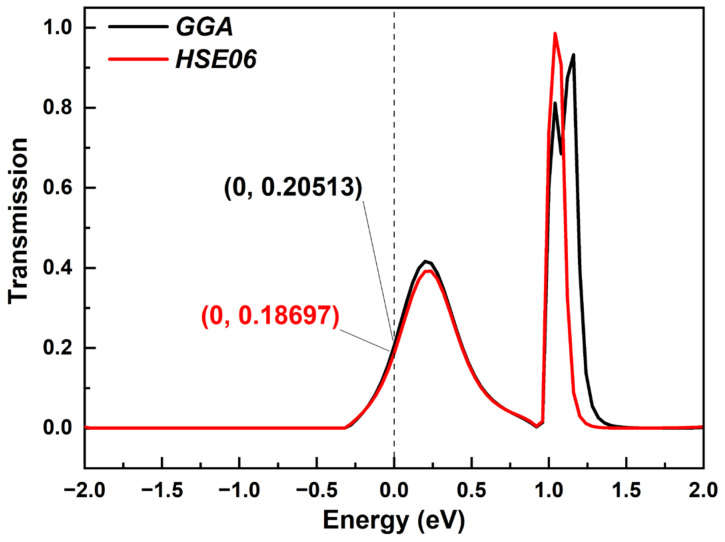
The comparison of electron transmission spectrum calculated with GGA and HSE06 functionals.

There is no doubt that DFT-GGA underestimates the band gap when calculating electron transmission. Here, we also chose the HSE06 function as a comparison. For device ring 1 as a representative, the calculated HOMO–LUMO gap was 0.2432472 eV with the GGA function, while the gap was 0.2700527 eV using the HSE06 function. The difference in the gap was relatively small. Furthermore, we also calculated the transmission spectrum around the E_F_ with the two functionals, which is shown in Figure 9. The electron transmission curves calculated with the two functionals are very close, especially for smaller energies, which is reflected in the similar transmission peaks and transmission coefficients. For larger energies, there were some little differences in the number and height of transmission peaks. Thus, there was a very small gap between the results calculated with GGA and HSE06 functionals. More importantly, such small differences did not affect the comparison between the molecular chains with different lengths, which was our research focus.

The conductance *G* can be expressed in terms of the transmission function within the Landauer–Büttiker formalism [47,48]:

(2) $$G=G_{0}\int_{-\infty }^{+\infty }dET\left(E,V\right)\left(-\frac{𝜕f\left(E\right)}{𝜕E}\right)$$

The current through a molecular junction is calculated from the Landauer–Büttiker equation [49]:

(3) $$I=\frac{2e}{h}\int_{-\infty }^{+\infty }dET\left(E,V\right)\left(f_{1}\left(E\right)-f_{2}\left(E\right)\right)$$

where *G*_0_ = 2*e*^2^/*h* is the quantum unit of conductance, *h* is the Planck’s constant, *e* is the electron charge, $f\left(E\right)$ is the Fermi distribution function, $f_{1,2}\left(E\right)$ are the Fermi functions of source and drain electrodes, and $T\left(E,V\right)$ is the quantum mechanical transmission probability of electrons, which can be given as [49]

(4) $$T\left(E,V\right)=tr\left[\Gamma_{L}\left(E,V\right)G^{R}\left(E,V\right)\Gamma_{R}\left(E,V\right)G^{A}\left(E,V\right)\right]$$

where *G^R^* and *G^A^* are the retarded and advanced Green functions of the conductor part respectively and $\Gamma_{L}$ and $\Gamma_{R}$ are the coupling functions to the left and right electrodes, respectively.

## 4. Conclusions

We have designed new C_2_N ring chains in different sizes and theoretically investigated comprehensive electron transport properties. The results show that the conductance of the new-type chain decayed exponentially with the length and so did the electron transmission. The length affected the conductance decay constant, which decreased when the length was greater than 4.5 nm. However, the electron tunneling mechanism still dominated the electron transport by the LUMO, which showed delocalized electronic states for each length. This new-type molecular wire showed one abnormal tunnel barrier energy-length curve, where shorter C_2_N ring chains with strong conductance exhibited higher barrier energy thanks to the compensation of their much narrower HOMO–LUMO gap. When a bias was applied, the currents of different wires grew at different rates. Furthermore, the NDR effect occurred in longer chains with an even number of C_2_N rings and increased with size. The reduction in the electron transmission peak brought about such a valuable effect. The two shortest C_2_N-ring-based chains showed peculiar conductance–voltage characteristics, which did not show any regularity in the length. Meanwhile, the six longer C_2_N-ring-chain-based devices presented regularly enhanced conductance with the voltage at larger rates for longer chains. The conductance attenuation speed and transport regime could be regulated effectively by applying the voltage. Furthermore, the new-type chain was demonstrated to be better than the parent material and other similar nanoribbon-based chains in electron transport. Some factors were also considered to investigate the corresponding influence on the new-type molecular chain. The common H impurity obviously enhanced the electron transmission of the C_2_N ring chain, while the common O impurity had a minimal effect on it. The structural disorder originating from the higher temperature did not change the exponential decreasing tendency of electron transmission with the length of the C_2_N ring chain and the electron transmission fluctuated with the temperature T within a small range when T did not exceed 400 K. The C_2_N ring chain with 1D GNR electrodes possessed noticeably stronger electron transmission than those with 2D graphene and 3D Au electrodes. These findings offer a solid backing for the application of C_2_N ring chains in tunneling diodes and controllable molecular devices.

## Data Availability

The data presented in this study are available on request from the corresponding author. The data are not publicly available due to the requirements of funding projects.

## Supplementary Materials

The following supporting information can be downloaded from https://www.mdpi.com/article/10.3390/molecules28247994/s1—Figure S1: Molecular electrostatic potential for the pore (unit cell) of C_2_N monolayer; Figure S2: (a,b) LUMOs and HOMOs of device C_2_N rings 5 and 6, respectively; Figure S3: (a,b) Transmission pathways of device C_2_N rings 4, 5, 6 and 7, respectively; Figure S4: (a–g) Comparison of electron transmission spectrum around E_F_ of the C_2_N ring chains with no defect and O and H impurities for device C_2_N rings 2, 3, 4, 5, 6, 7 and 8, respectively; Figure S5: (a–e) I–V curves of device C_2_N rings 2, 3, 5, 6 and 8, respectively. Each inset shows the first derivative of the corresponding I–V curve at biases, representing the growth rate of the current with the bias; Figure S6: (a–h) Transmission spectrum within wider energy ranges at 0 V, 0.1 V, 0.2 V, 0.3 V and 0.4 V of device C_2_N rings 1–8, respectively; Figure S7: (a–d) Transmission spectrum within narrower energy ranges at 0 V, 0.1 V, 0.2 V, 0.3 V and 0.4 V of device C_2_N rings 2, 4, 5 and 7, respectively; Figure S8: (a,b) Conductance as a function of number of C_2_N rings with an exponential fit at 0.2 V and 0.3 V, respectively. Each inset exhibits the corresponding lnG as a function of length with a linear fit.
