# Supplementary material for: Size- and Voltage-Dependent Electron Transport of C2N-Rings-Based Molecular Chains
Source: Molecules. 2023 Dec 7;28(24):7994. doi: 10.3390/molecules28247994 (PMC10745836; doi:10.3390/molecules28247994)
Supplement: Supplementary file 1 [file molecules-28-07994-s001.zip › Supporting Information-12-7.pdf]

Supporting Information for Size- and Voltage-Dependent Electron Transport of C_2N-Rings-Based Molecular Chains

Dian Song [1], Jie Li [1,*], Kun Liu [1], Junnan Guo [2], Hui Li [2,*] and Artem Okulov [3]

[1] School of Materials Science and Engineering, Jiangsu University of Science and Technology,
Zhenjiang 212100, China; sssdian2001@163.com (D.S.); liu_kun@163.com (K.L.)

[2] Key Laboratory for Liquid-Solid Structural Evolution and Processing of Materials, Ministry of Education, Shandong University, Jinan 250061, China; guojunnan113005@hotmail.com

[3] M.N. Mikheev Institute of Metal Physics, Ural Branch of Russian Academy of Sciences,
Ekaterinburg 620077, Russia; okulovartem@imp.uran.ru

* Correspondence: jie_li@just.edu.cn (J.L.); lihuilmy@sdu.edu.cn (H.L.)

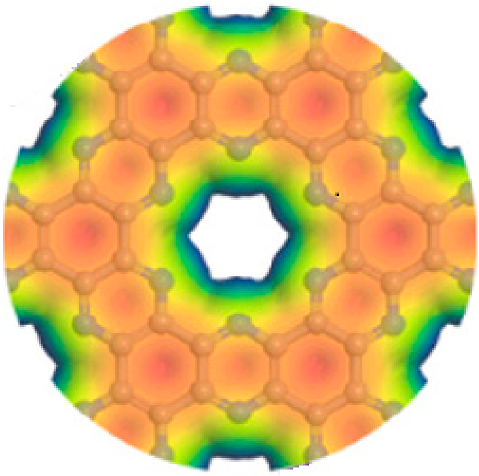

Figure S1 Molecular electrostatic potential for the pore (unit cell) of C_2N monolayer [17].

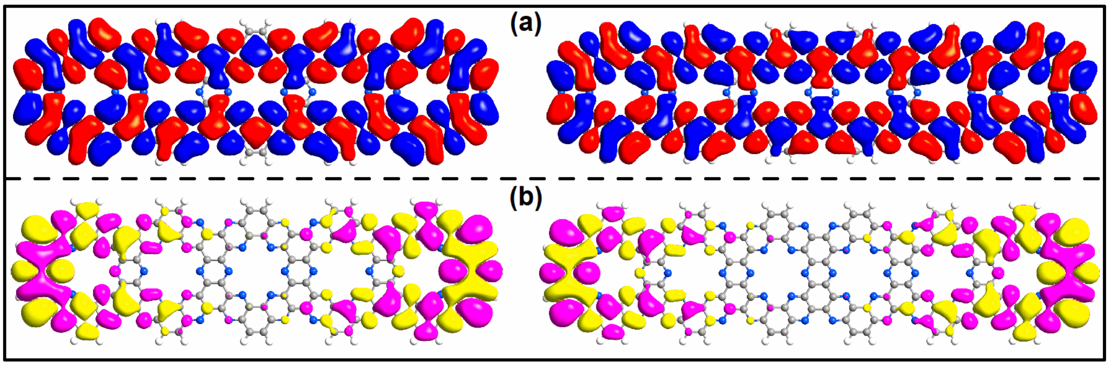

Figure S2 (a-b) LUMOs and HOMOs of Device C_2N ring5 and 6, respectively.

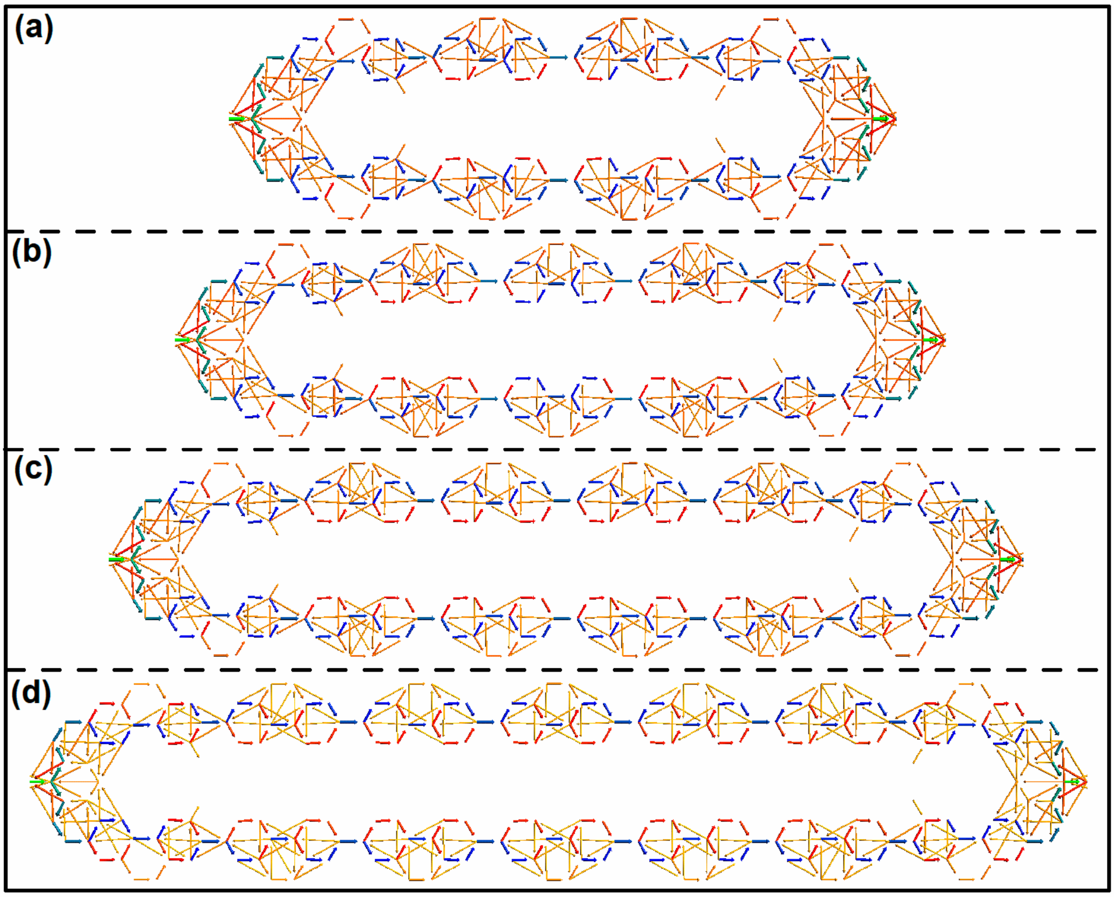

Figure S3 (a-b) Transmission pathways of Device C_2N ring4, 5, 6 and 7, respectively.

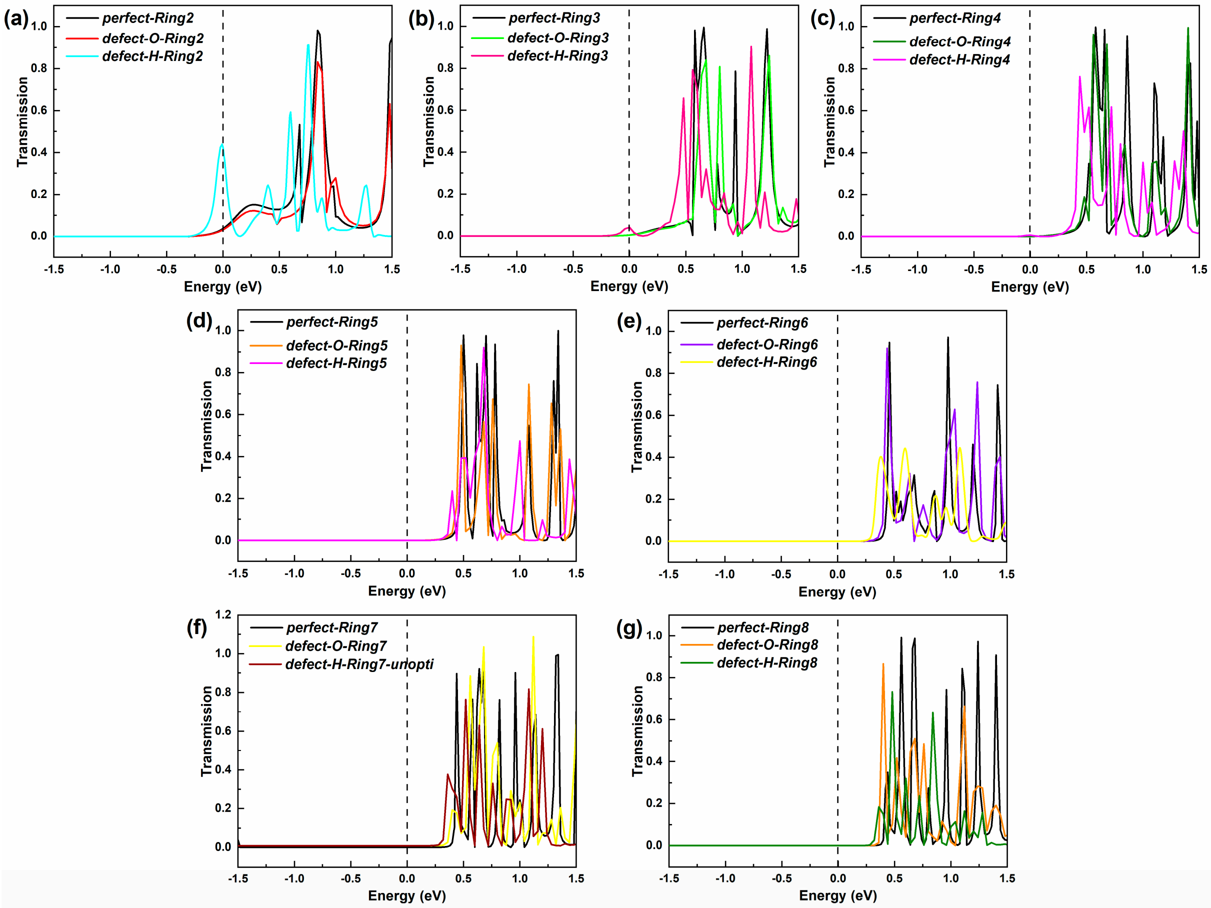

Figure S4 (a-g) Comparison of electron transmission spectrum around E_F of the C_2N rings chains with no defect, O and H impurities for Device C_2N ring 2, 3, 4, 5, 6, 7 and 8, respectively.

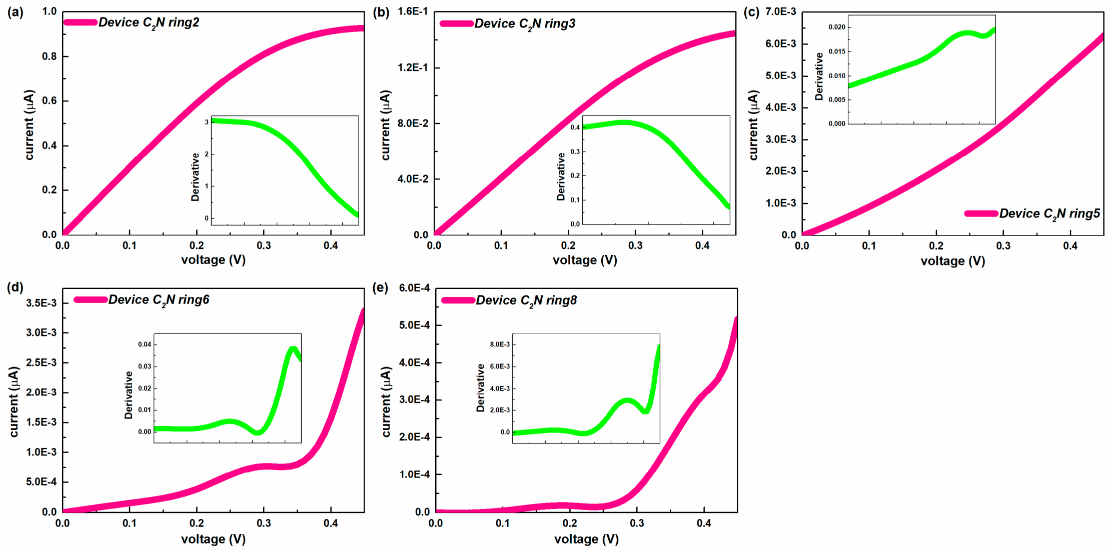

Figure S5 (a-e) I-V curves of Device C_2N ring2, 3, 5, 6, 8, respectively. Each inset shows the first derivative of corresponding I-V curve at biases, representing the growth rate of the current with the bias.

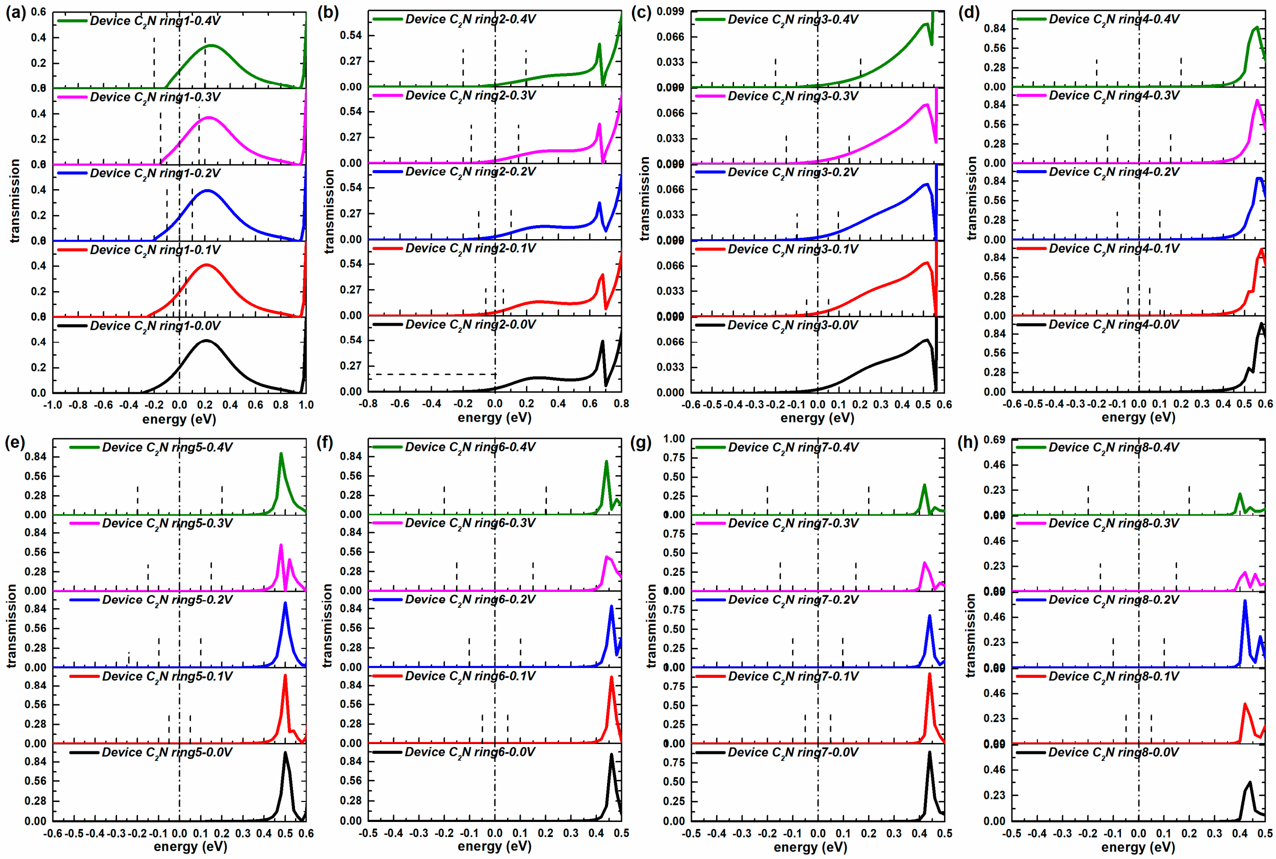

Figure S6 (a-h) Transmission spectrum within wider energy ranges at 0V, 0.1V, 0.2V. 0.3V and 0.4V of Device C_2N ring1-8, respectively.

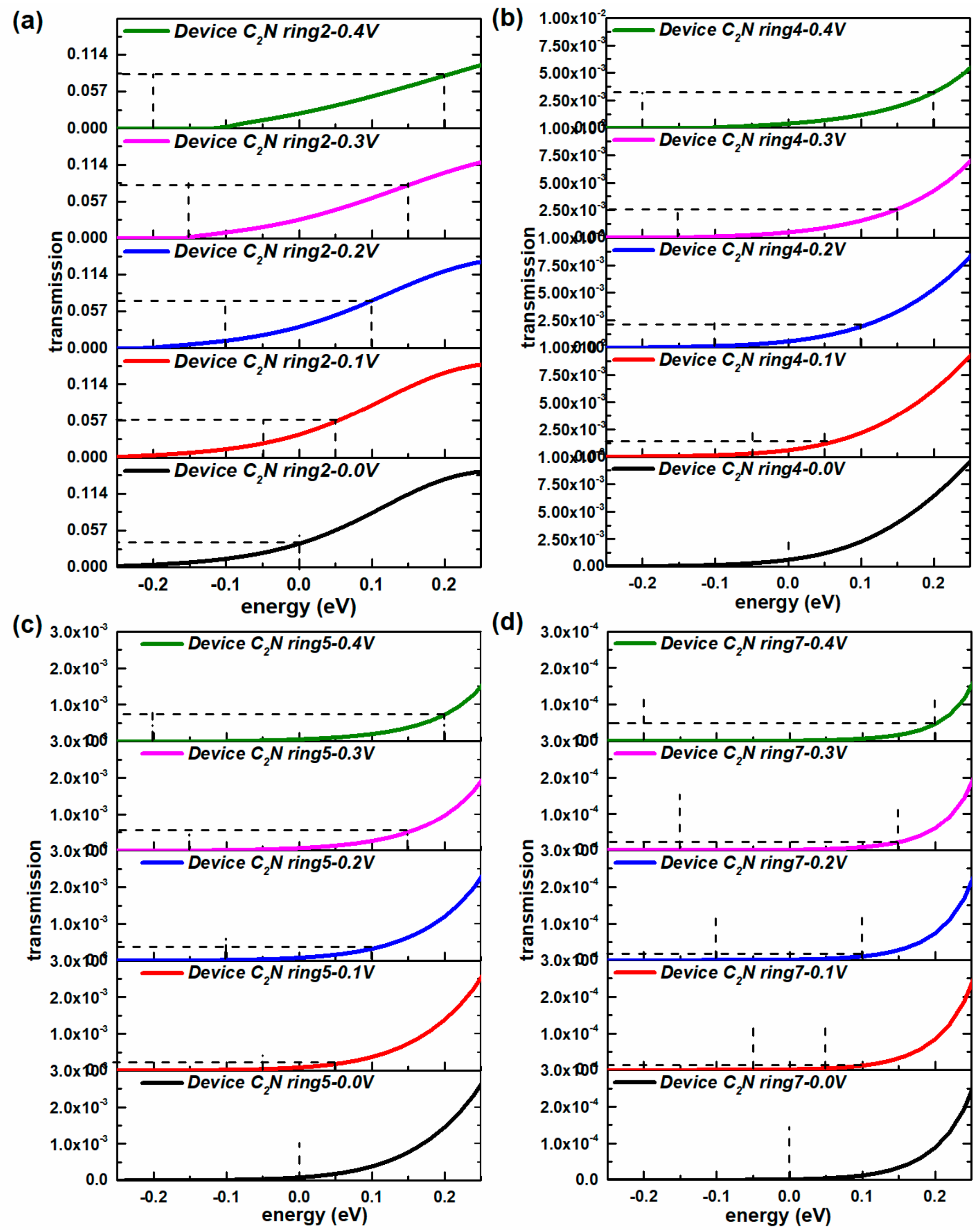

Figure S7 (a-d) Transmission spectrum within narrower energy ranges at 0V, 0.1V, 0.2V. 0.3V and 0.4V of Device C_2N ring2, 4, 5 and 7, respectively.

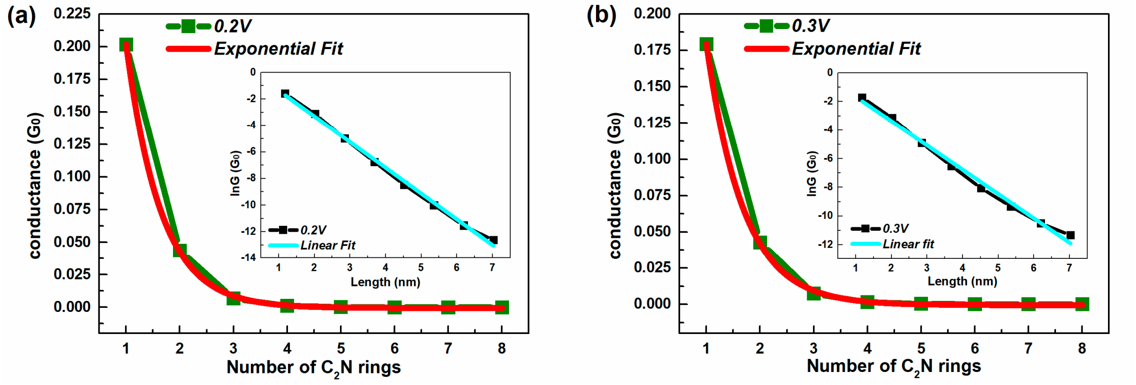

Figure S8 (a-b) Conductance as a function of number of C_2N rings with an exponential fit at 0.2V, and 0.3V, respectively. Each inset exhibits the corresponding lnG as a function of length with a linear fit.
