# Supplementary figures and images for: Size- and Voltage-Dependent Electron Transport of C2N-Rings-Based Molecular Chains
Source: Molecules. 2023 Dec 7;28(24):7994. doi: 10.3390/molecules28247994 (PMC10745836; doi:10.3390/molecules28247994)

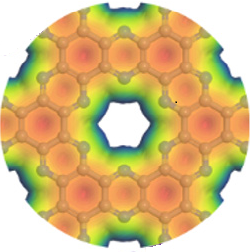

Supplement: Supplementary file 1 [file molecules-28-07994-s001.zip › SP-1.bmp]
